# On the determination of the temperature distribution within the color conversion elements of phosphor converted LEDs

**DOI:** 10.1038/s41598-017-10114-6

**Published:** 2017-08-30

**Authors:** Wolfgang Nemitz, Paul Fulmek, Johann Nicolics, Frank Reil, Franz P. Wenzl

**Affiliations:** 1 Institute of Surface Technologies and Photonics, JOANNEUM RESEARCH Forschungsgesm.b.H., Franz-Pichler-Straße 30, A-8160 Weiz, Austria; 2 Institute of Sensor & Actuator Systems, TU Wien, Gusshausstraße 27-29, A-1040 Vienna, Austria

## Abstract

We present an iterative optical and thermal simulation procedure which enables the determination of the temperature distribution in the phosphor layer of a phosphor converted LED with good accuracy. Using the simulation both the highest phosphor temperatures, which are mostly relevant to material degradation as well as the temperatures of those phosphor particles which mainly contribute to converted light emission can be determined. We compare the simulations with experimental studies on the phosphor temperature. While infrared thermography only gives information on the phosphor layer surface temperature, phosphor thermometry provides temperature data on the volume temperature of the phosphor layer relevant to color conversion.

## Introduction

Even though the last few years have witnessed an increasing market penetration of solid state lighting sources, light-emitting diode (LED) based lighting solutions are a long way from reaching their full potentials^1^. Despite their superior energy efficiency, concerns regarding white light quality impede the replacement of traditional lighting sources with LED-based luminaires. Phosphor converted (PC)-LEDs are based on the combination of typically blue LED light and the excited emission from one or more phosphor materials embedded in a matrix material such as silicone. These phosphor materials determine spectral power distribution and color rendering index^2, 3^ of the white light emitted. Still, the shape and the arrangement of the color conversion elements (CCEs, phosphors in a matrix material) also strongly influence white light quality and output^4–10^. For instance, a geometrical mismatch between CCE and LED die may result in anisotropic color distribution, like a bluish center and a yellowish halo or vice-versa^4^. Another challenge is correlated color temperature (CCT) stability as a result of temperature changes during operation (e.g., at elevated current and/or temperature levels^11^) or material degradation^12, 13^.

For general lighting applications, it has been suggested that color variations among individual LEDs should be confined to a 2-step MacAdam-ellipse^14^. This turned out to be a major challenge for various reasons. One important issue is the high thermal load of a CCE during operation, in particular of those with phosphor particles in a silicone matrix^15–23^. This is related to the low thermal conductivity of silicone. The negative correlation of the phosphor’s quantum efficiency with temperature results in a relative decrease of the yellow-converted light intensity at elevated temperatures. This is why a multipronged initiative in phosphor research is proposed in the solid state lighting R&D plan of the US Department of Energy^24^. The goal is to reduce the current state-of-the-art phosphor quantum efficiency drop of 10% to 5% by 2020, for a temperature increase from 25 °C to 150 °C. Also, novel design considerations based on information on the local temperatures within a CCE during LED operation are essential in order to efficiently counteract a shift in the CCT value. However, obtaining such information is all but trivial. As discussed in a recent manuscript^25^, the determination of the phosphor temperature has been a challenging problem for years. Thermo-couple measurements turned out to be inconsistent^26^ and infrared thermography is restricted to the CCE surface but not the relevant bulk temperature^25^. Cutting a LED package open^18^ turned out to be too disruptive to allow meaningful measurements for good agreement between simulation and experiment. Also some approaches based on simulations have been reported to have their limitations with respect to the local phosphor temperature^25^.

In our recent studies, we used a combination of optical and thermal simulations in order to highlight the large thermal load of CCEs^20, 21, 27^. However, in these studies the quantum efficiency of the phosphor was kept constant at its room temperature value. This means, no drop of quantum efficiency with temperature was considered in these simulations.

In reality though, a drop in quantum efficiency with temperature leads to even higher temperatures of the CCEs and finally a thermal runaway^28^. The refractive index n of silicone changes with temperature as well. Typically, n and the thermo-optic coefficient dn/dT of silicone can be adjusted by the choice of its side-groups and its cross linking densities^29^. This way, dn/dT can be set to values between −1.5 × 10^−4^ K^−1^ and −5 × 10^−4^ K^−1^ ^30^. The thermo-optic coefficient of phosphors like Ce:YAG is about two orders of magnitude lower^31^. This leads to an increasing refractive index mismatch of both materials with increasing temperature^32^, resulting in stronger light scattering and absorption of blue light within the CCE. In essence, our recent studies only highlighted some general coherences of the thermal load of CCEs and gave only a lower bound estimate on the temperature.

In the present study we continue our previous work with an iterative method of optical and thermal simulations, which includes temperature dependence of material parameters. These simulations predict the local temperature distribution within the CCE volume and match measured data on LED modules much more closely. In particular we discuss the temperature values of those regions within the CCE, which are most relevant for the color conversion process and which determine the temperature dependent color shifts of phosphor converted LEDs under operation. In addition, we juxtapose results from infrared thermography, a method usually applied to determine the temperature of a CCE^18, 19, 22, 23^, with those from phosphor thermometry. The latter turns out as a promising method to solve the open question on the experimental determination of the relevant phosphor temperature of phosphor converted LEDs under operation.

## Experimental

### Optical Simulation

The details of the optical simulation procedure can be found in a previous publication^4^. It is based on a suitable simulation model comprising a blue LED die with a CCE on top of it. The die is placed on a printed circuit board (PCB) by chip-on-board technology. The calculations were performed with the commercial ray-tracing simulation software package ASAP.

Two wavelengths are considered, representing the blue excitation LED light (460 nm) and the converted yellow light (565 nm). We assume that only the blue LED light is absorbed by the phosphor particles, amounting to an extinction coefficient of zero for yellow light and 10^−3^ for blue light. Both blue and yellow light are scattered according to the Mie scattering model. The phosphor particles have a mean radius of 7.8 µm and a standard deviation of 4.2 µm. Their concentration within the silicone matrix is 11.5 vol.%. The refractive indexes of silicone (used for CCE and adhesive layer) and phosphor are 1.4 and 1.63 for both wavelengths at 25 °C. The thermo-optic coefficient of the silicone was determined from ellipsometry measurements of a thin silicone layer at different temperatures as dn/dT = −2.9 × 10^–^^4^ K^−1^, the coefficient for the phosphor is assumed to be negligible.

In order to obtain its absorption profile, the CCE is divided into a number of voxels. The absolute amount of blue radiant flux per voxel is the input parameter for the thermal simulations^20^.

### Thermal Simulation

As discussed in previous publications^20, 21, 27^, the spatially resolved absorbed flux of the blue LED light within the CCE is taken as input parameter for the subsequent thermal simulations using the GPL-software packages GetDP/Gmsh (Finite Element Method, FEM). Calculation effort and time are reduced by taking advantage of symmetry considerations and simulating only one-eighth of the whole LED package. Adhesive layer and CCE are modelled as blocks with individual thermal conductivities and heat capacities.

The two heat sources in the thermal simulations are the power loss of the LED die as determined by electro-thermal simulations, and contributions from the absorbed blue light. The latter is mainly comprised of contributions from the Stokes’ shift (blue to yellow) and from non-radiative recombination (quantum efficiency smaller than unity).

The following boundary conditions are defined: The bottom surface of the printed circuit board is assumed to be mounted on a perfect cooler of constant temperature T_cool_ (Dirichlet boundary condition). All other boundaries of the model are subject to ambient air convection at 300 K and h = 20 W/(m²K)^20^. The CCE thermal conductivity is set at 0.27 W/mK as determined by a procedure discussed in^ 20^.

The FEM is based on well-established algorithms to solve systems of linear equations: A*T = B, with the stiffness matrix A of the problem, the temperature vector T to be determined and the source term B. A temperature dependence of thermal conductivities or of heat sources lead to a non-linear system of the form A(T)*T = B which has to be solved iteratively. Convergence can be obtained, e.g., by using the Newton-Raphson method, which additionally uses a Jacobian-matrix (d/dT)(A(T)*T). This Jacobian-matrix is constructed for each iterative step by using a linearization of the nonlinearity at the previous solutions for T.

### Measurements

The CCE was fabricated from thin films of a phosphor-silicon slurry with a phosphor concentration of 11.5 vol.%. The phosphor is a Eu^2+^ doped orthosilicate^33^ (from an older batch provided by Tridonic Jennersdorf GmbH in the course of some previous joint research projects. Phosphor related parameters were taken from the respective data sheet). The thickness of this foil was measured from cross-sections of the finished LED module. Phosphor tiles matching the size and shape of the LED die were cut out from the thin films using a femtosecond laser. The fabricated tiles were a bit smaller (936 µm) than initially defined (940 µm). This was due to the cutting width of the laser cutting process. This way a number of identical phosphor tiles can be created, which can be mounted on the LED die by a gluing layer and which, e.g., can be applied to measure the impact of the thickness of the gluing layer. The latter again can be determined by cross-sections of the module (taken as a mean value, phosphor tiles were glued by hand with some µm variation of the gluing layer thickness along the chip surface). From these measurements the geometric parameters as input for the simulation model described in the previous paragraphs were deduced. The thermo-optic coefficient of the silicone was determined using ellipsometry measurements of a pristine silicone layer at different temperatures. These measurements served as inputs for the simulation model described in the previous paragraphs.

To verify the simulations, thermograms were taken using the high resolution IR thermography system Infratec IR8300 which is sensitive between 3 and 5 µm. The LED module was attached to a copper block. The temperature of this block was controlled by a Pt100-sensor and a Peltier element to adjust the bottom temperature of the module to 25 °C during the measurement. As a prerequisite to deduce temperatures from thermography experiments, the emissivity of the measurement object had to be determined by a series of isothermal experiments.

In phosphor thermometry, a calibration of temperature and decay time must first be performed. For this, a thin silicone film with phosphor particles was placed on a hot plate (Stuart CB300) at a number of temperatures. Before each measurement of the phosphor decay time, the temperature was kept constant for 15 minutes. Also, the sample was shielded from the ambient air flow with a box. A function generator (HP 8116 A) connected to a self-made voltage-current-converter provided a square signal current to power an LED (Kingbright L-7104QBC-D) as an excitation light source. The LED was chosen because of its fast switching capability. The fluorescent light was collected with a glass fiber and guided through a low pass, to suppress the excitation light, to a photo multiplier (Hamamatsu H10721-01). The photocurrent transformed into a corresponding voltage was measured by an oscilloscope (Agilent 54831D, Infiniium). The oscilloscope trigger was controlled by the function generator, and the measured signal averaged over up to a thousand times to improve the signal-to-noise ratio.

For the measurements of the emission decay time of the fabricated CCEs on top of an operating LED at 500 mA bias current, that LED module was mounted on the same copper block as for the infrared thermographic measurement to keep the PCB bottom surface temperature at 25 °C.

The datasets generated and/or analyzed in the current study are available from the corresponding author upon reasonable request.

## Results and Discussion

The simulation model consists of a square-shaped CCE with a flat surface attached to a blue emitting LED die by an adhesive layer. The CCE consists of orthosilicate based phosphor particles embedded in a silicone matrix.

**Figure 1:**
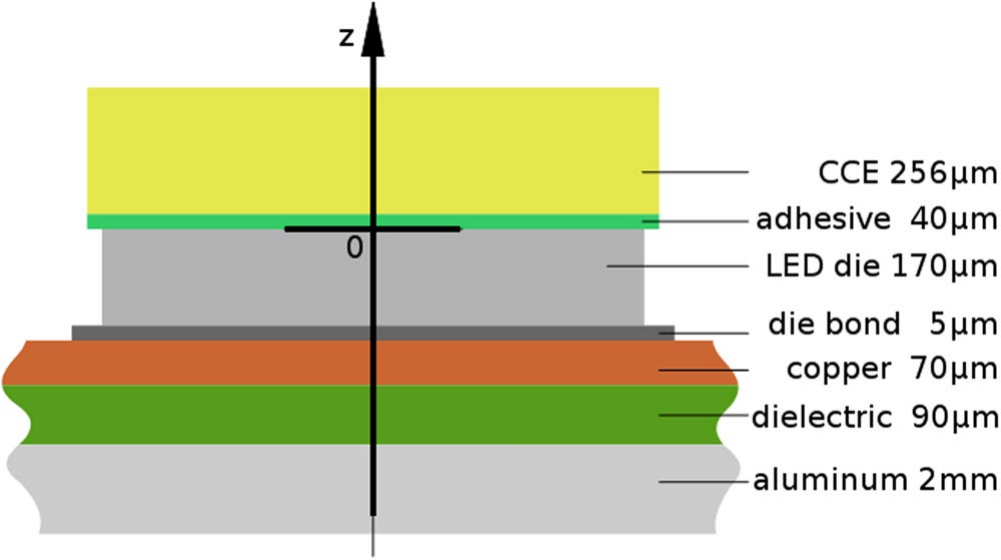
The simulation model consists of a blue emitting LED die and a square-shaped CCE placed on top of the die with the help of an adhesive layer (40 µm) of pristine silicone. The die has a height of 170 µm and lateral dimensions of 880 × 880 µm^2^, whereas the dimensions of the active layer are 850 × 850 µm^2^. The lateral dimensions of the CCE are 936 × 936 µm^2^. The height of the CCE is 256 µm. The vertical direction is defined as z-direction of the module. The LED die is mounted on a printed circuit board with the help of a die bond layer (5 µm). The printed circuit board consists of a copper layer, a dielectric layer and an aluminum layer.

**Figure 2:**
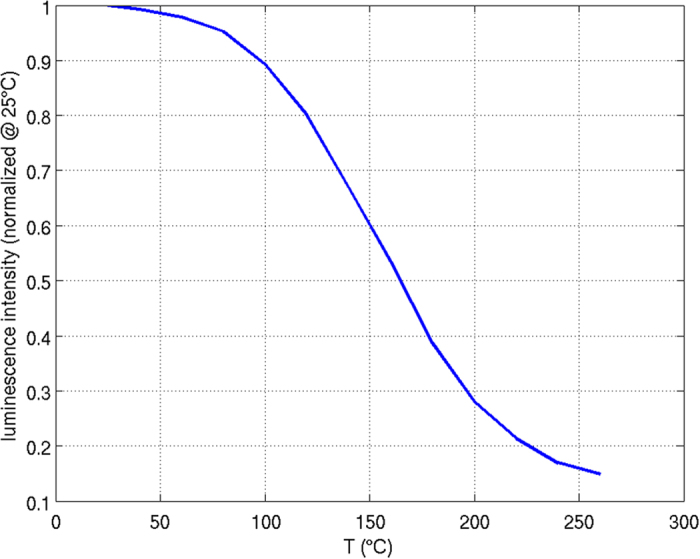
Experimentally determined temperature dependence of the luminescence intensity of the orthosilicate phosphor used in this study (normalized to the intensity at 25 °C).

The dimensions of the blue LED die are 880 × 880 × 170 µm³ (W × L × H) with an active layer of 850 × 850 µm^2^ (Cree EZ900, Gen II). The die itself is mounted on a PCB by an adhesive layer with a thickness (here) of 5 µm. The CCE (936 × 936 µm^2^) is glued onto the LED die by a 40 µm thick adhesive layer of pristine silicone with the same lateral dimensions as the CCE. The height of the CCE is 256 µm. Figure 1 demonstrates the set-up of the LED module. Both the height of the adhesive layer and the kind of phosphor material were chosen to induce a notable thermal load on the CCE to facilitate the study of thermal effects. For instance, orthosilicate based phosphors show a more pronounced temperature dependent drop in luminescence than phosphors like Ce:YAG^34^. Figure 2 shows the measured temperature dependence of the phosphor luminescence intensity, normalized to its intensity at 25 °C. These data are in agreement with other studies on orthosilicate based phosphors^35^.

**Figure 3:**
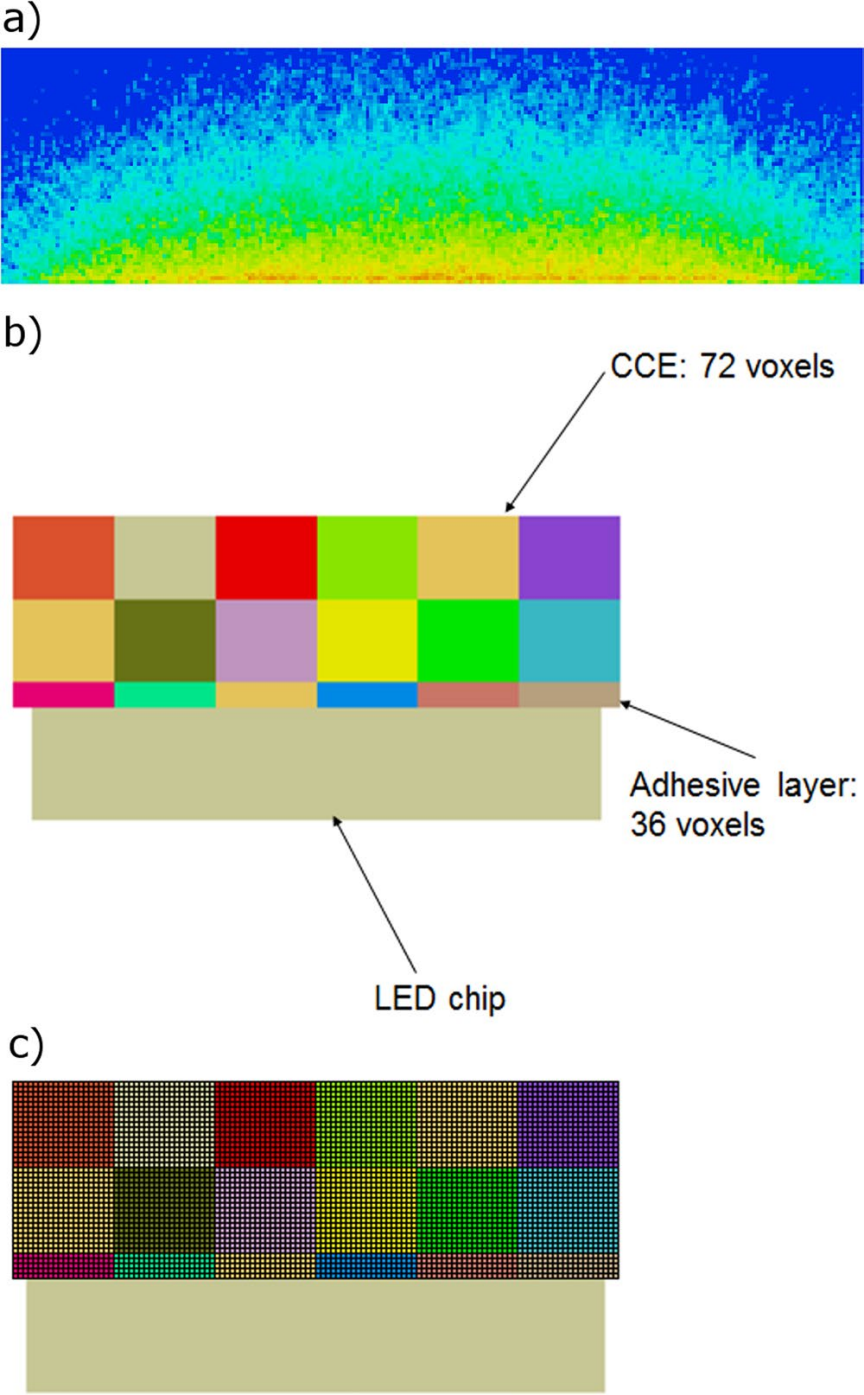
(**a**) Absorbed intensity distribution of the blue radiant flux within the CCE. The reddish color depicts the areas of highest absorbed intensity. This distribution is determined by dividing the CCE into a number of voxels, each having a size of 4 × 4 × 4 μm³. Most of the absorption (and therefore also recombination) processes take place close to the LED die surface. (**b**) In order to consider the temperature dependency of the refractive index of the silicone, the CCE is divided into 72 voxels, each having a size of 156 × 156 × 138 μm^3^. For each of these voxels, the refractive index is defined in accordance with the local temperature and the thermo-optic coefficient of the silicone (mean value of the temperature along the voxel). The local temperature is determined by the thermal simulations. (**c**) Sketch of the voxel distributions for the determination of the absorbed intensity distribution of the blue radiant flux (4 × 4 × 4 μm^3^) and for the variation of the refractive indexes (156 × 156 × 138 μm^3^). For all the voxels used to determine the blue radiant flux that are located within a voxel that determines the refractive index the same refractive index is taken.

As in our previous studies^20, 21, 27^, the spatial absorption profile of the blue LED light within the CCE (see Fig. 3a) is used as an input for the subsequent thermal simulations. Here, the CCE is subdivided into a number of small voxels of 4 × 4 × 4 μm³. For each of these voxels the absorbed intensity of blue LED light is determined for a given blue radiant flux, which is determined from the related data sheet as a function of the applied current^36^. Using this, the number of absorption processes of the blue LED light by the phosphor particles can be determined spatially resolved. Since these absorption processes go hand in hand with subsequent recombination processes, the local heat generation can also be determined within the CCE with the same spatial resolution. In each iteration step, the optical and thermal simulation algorithms require repeated mutual exchange of interdependently varying parameters. For example, the optical simulation translates the temperature distribution provided by the thermal simulation into a distribution of refractive indexes of the silicone within the CCE, updates the light absorption distribution in the CCE and feeds the new data back to the thermal simulation. The thermal simulations then generate temperature distributions from these data, taking into account the heat generation, depending on quantum efficiency and Stokes shift. Figure 3b illustrates the voxel configuration for the consideration of refractive index variations. It needs to be noted that, due to the large number of boundary conditions stemming from the different refractive indexes of each voxel, the number of refractive index voxels was kept lower (72 voxels, each 156 × 156 × 138 μm³) than for the intensity distribution of the blue radiant flux (4 × 4 × 4 μm³). This means that for all the voxels used to determine the blue radiant flux that are located within a voxel that determines the refractive index the same refractive index is taken. A schematic of this is shown in Fig. 3c.

These iterations stop once the convergence criteria is met, which is the case when the difference of two successive steps falls below a predefined value.

**Figure 4:**
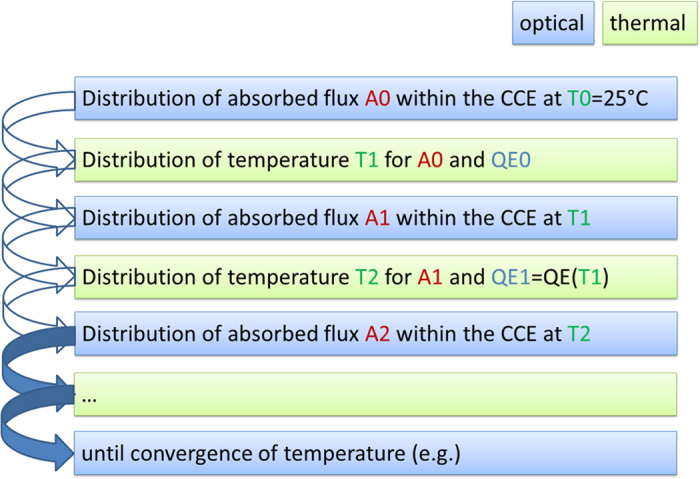
Sketch of the iterative optical and thermal simulation procedure.

Figure 4 shows the schematic of the iterative simulation procedure in detail. The procedure starts with the determination of the distribution of blue light absorption within the CCE at 25 °C (room temperature values of the optical parameters). Subsequently, the thermal simulation delivers the first temperature distribution which is translated into a refractive index distribution which in turn is the basis for the calculation of an updated distribution of blue light absorption. This new absorption distribution is again the base for a thermal simulation step, which takes into account the luminescence intensity at the given temperature. Note, that the loss of luminescence intensity (see Fig. 2) has been equated with a loss of quantum efficiency. For a more detailed study it should be considered, that the overall loss of luminescence intensity to some fraction also may be caused by a temperature dependent modification of the extinction coefficient.

**Figure 5:**
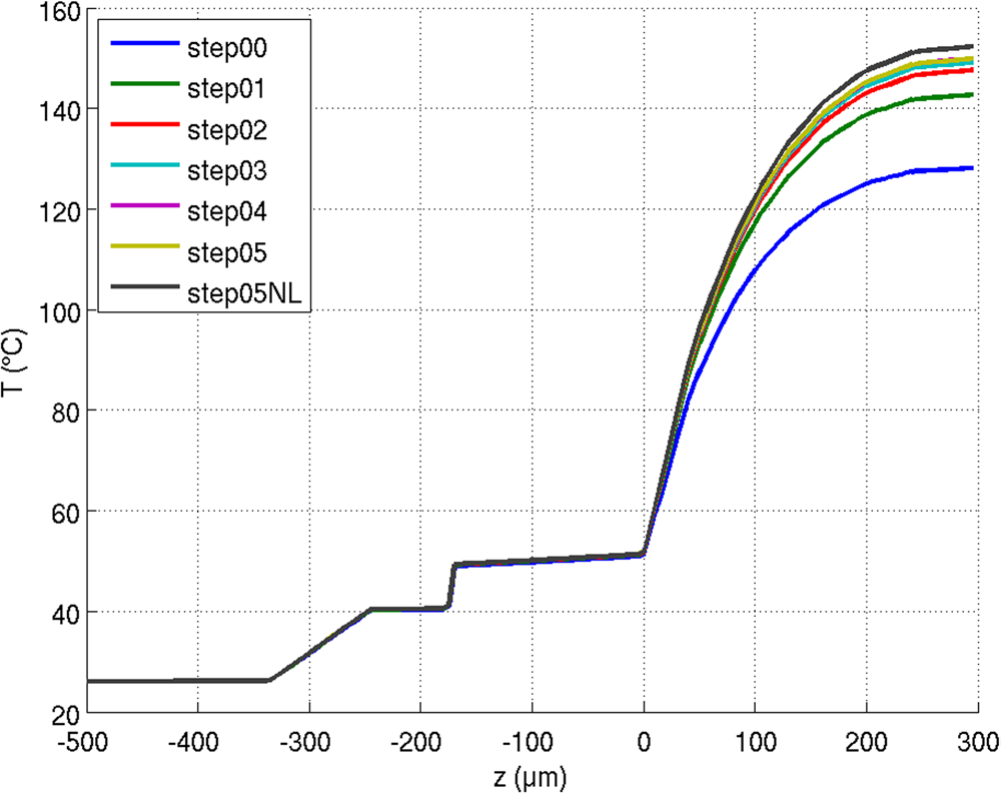
Temperature distributions for the individual iterative simulation runs till two subsequent temperature distributions converge (in this case after 6 simulation steps). The temperature distribution is shown along the z-direction, the CCE starts at 40 μm. For the last simulation run, the simulations were also performed considering a temperature dependence of the thermal conductivity ﻿(step05NL).

Figure 5 illustrates the convergence criterion for an LED module at 500 mA operating current. The figure depicts the temperature distribution of the module (z-direction in Fig. 1). The bottom side of the adhesive layer for the CCE is located at a height of 0 µm. After 6 iterations the temperature distributions have largely converged, resulting in a final temperature of about 150 °C at the CCE surface. Also shown is a final simulation, in which the last thermal simulation step is repeated and in which a temperature dependence of the CCE’s thermal conductivity is considered. Here, a temperature coefficient of −0.004 1/K for silicone was chosen. This value was taken from a study by Moreira *et al*.^37^ and extrapolated to higher temperatures. As a result, a final temperature of 152.3 °C is reached at the surface of the CCE.

**Figure 6:**
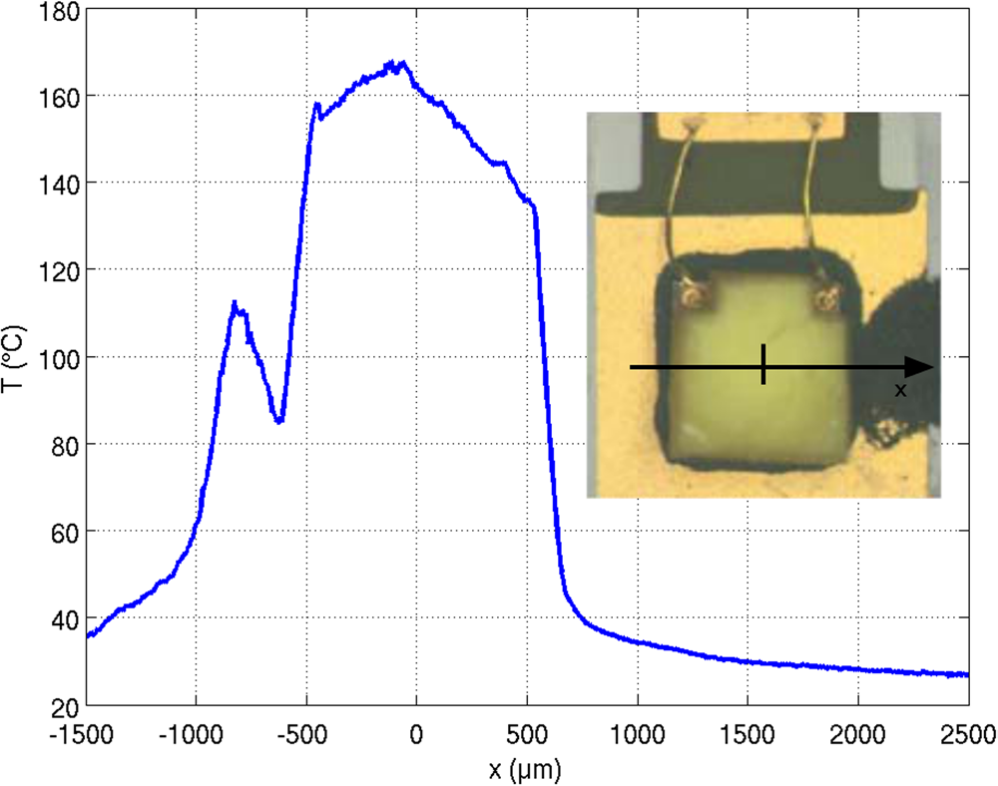
Result of the emission-corrected thermography measurement for the LED package at a forward current of 500 mA. The inset shows a microscope image of the LED package.

These simulations were compared with the performance of fabricated samples. Figure 6 shows an infrared thermographic line scan at an LED current of 500 mA. Here, a maximum temperature of about 163 °C was measured. While there are still small temperature differences between measurement and simulation, the simulation data, which include temperature dependence of refractive index and quantum efficiency, agree much better with reality than those which do not consider these temperature dependencies (cp. the first temperature distribution in Fig. 5 for a surface temperature of about 125 °C). Further improvement of the simulation may include a more delicate distinction between the temperature dependence of quantum efficiency and extinction coefficient and a consideration of temperature dependent wavelength shifts of the blue LED emission.

**Figure 7:**
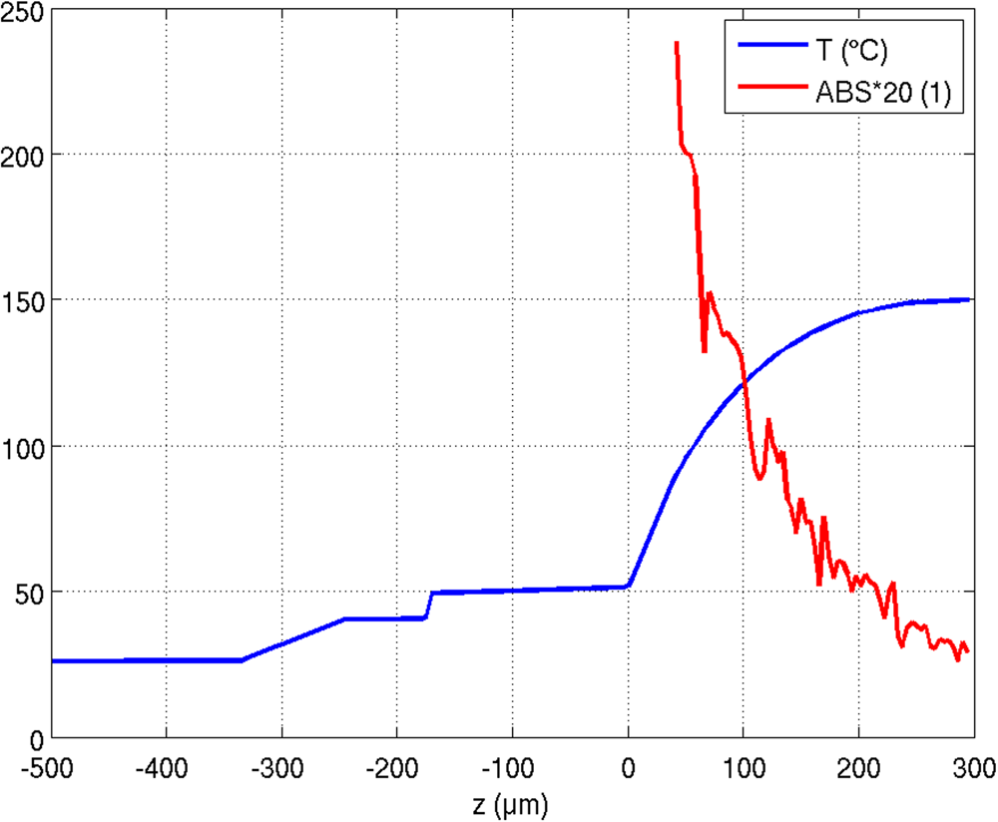
A comparison of the temperature distribution and the absorption profile of the blue LED light in the CCE. The CCE starts at 40 μm.

Still, the iterative thermal and optical simulation method presented here allows the determination of the volume resolved temperature distribution within the CCE while infrared thermography is restricted to the surface. It needs to be kept in mind, that most of the blue LED light is absorbed and converted into yellow light close to the LED die surface. This can be deduced from Fig. 3 and is shown in more detail in Fig. 7, which displays the temperature distribution of the final thermal simulation run together with the absorption profile of the blue LED light in the CCE (starting on top of the adhesive layer). Although most of the converted light and therefore heat is generated in the bottom part of the CCE, this heat can be dissipated more easily to the close-by LED die, which acts as a heat sink. Therefore, in reality, the temperature in the immediate vicinity of the LED die is most relevant for the amount of converted light generation and therefore the overall emission spectrum and the temperature dependent color shift. This important temperature is not directly measureable using infrared thermography (cp. Fig. 7).

A similar problem occurs in cancer photo-thermal therapy as discussed in^38^: efficient tumor destruction in animals usually requires local heating to a specific temperature, where cancer cells are destroyed. This requires accurate real time monitoring of the tumor temperature during the treatment^38^ in order to minimize collateral damage of healthy surrounding tissue. However, as reported by the authors of the respective study, infrared thermography, which is often applied in this regard, suffers from the drawback that it only provides information on the surface temperature (just at the skin level) while the actual temperature of subcutaneous tumors could significantly differ^38^. As discussed by the authors, luminescence nanothermometry could overcome this problem; in this case the authors used fluorescent nanoparticles that acted as local thermal sensors. Their fluorescence signature provided information on the temperature of the subcutaneous tumor level^38^.

For phosphor converted LEDs the most straight-forward way would be therefore also to use the phosphor emission itself as a temperature indicator. This could be done using phosphor thermometry, a technique that, besides in a study by Zukauskas *et al*.^39^, has not been frequently used in LED technology yet. In particular, it would provide the information on the temperature directly from those regions inside the CCE, in which most of the color conversion processes take place and which therefore is also most relevant for the final spectral power distribution of white light emission.

**Figure 8:**
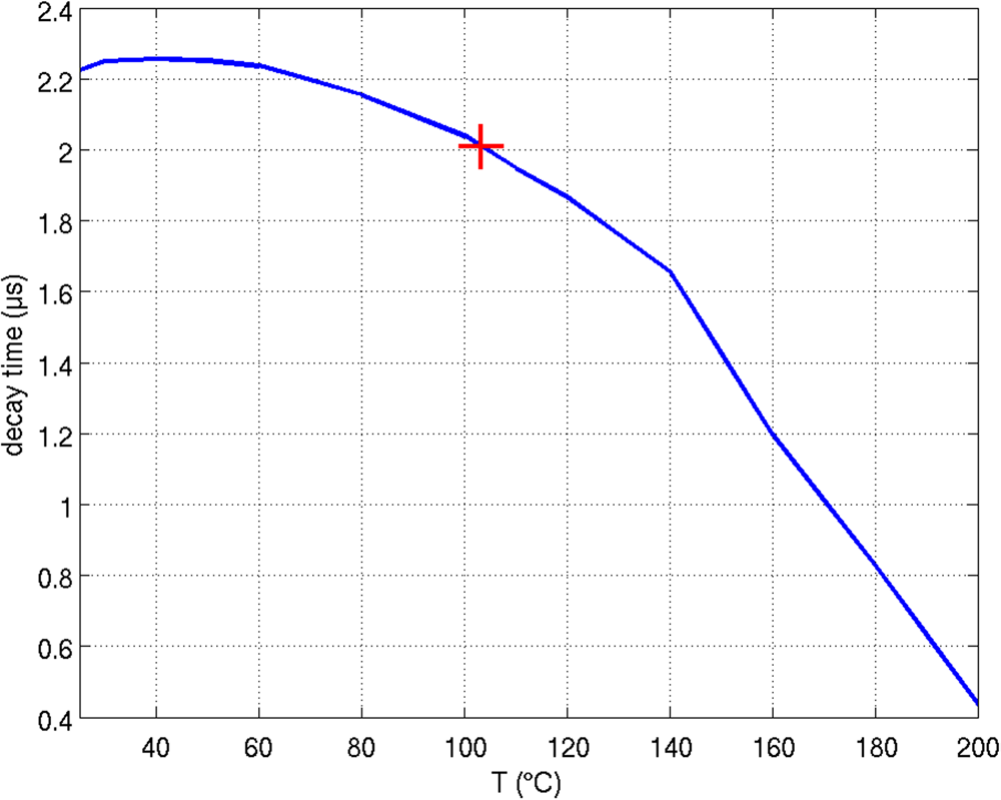
Decay times of a phosphor silicone layer and the emission from the LED as determined by phosphor thermometry. The LED emission shows a decay time of about 2.02 μs, which corresponds to a temperature of about 104 °C.

To study the applicability of such an approach, we fabricated very thin films of phosphor in silicone and put them on a hot plate. The phosphor’s decay time was measured as a function of temperature, see the blue curve in Fig. 8. These values are taken as a reference in order to assign the decay time of the converted light from the LED under operation at 500 mA to the temperature the phosphor is exposed to. In particular, since in this case the decay time of the converted light is measured, the method allows to measure the temperature the phosphor is exposed to in that portions of the CCE that mostly contribute to the converted light emission. From this decay time, which was measured to be 2.02 μs, we determine a phosphor temperature of about 104 °C for those phosphor particles that mainly contribute to the generation of converted light. This is in good accordance with the simulated temperature in a distance of about 50–60 μm from the LED die surface (note that the CCE starts at a distance of 40 μm from the LED die surface). In accordance with Fig. 3a, this is the area in which most of the absorption processes of the blue LED light and therefore also color conversion processes take place.

Therefore, the temperature distribution throughout the CCE can be divided into two regimes, one close to the CCE surface, where, at least for the present CCE geometry and composition, the highest temperatures prevail and which is therefore most relevant with respect to materials degradation. The second regime close to the LED die surface provides information on the temperature values those phosphor particles are exposed to, which mainly contribute to converted light emission. Therefore, in particular this temperature regime should be taken into consideration for an estimation of temperature induced color shifts of phosphor converted LEDs. Both temperature regimes can be determined by the iterative optical and thermal simulation procedure described.

The relevance with respect to these two regimes becomes even more evident, when one considers the two temperature levels of 152.3 ° C and 104 °C measured at the surface and from the area most relevant to color conversion. Comparing this with Fig. 2, these two temperature levels correspond with a difference in quantum efficiency of about 30%. In a recent study we have investigated the impact of variations of the quantum efficiency of the phosphor on color shifts of the emitted white light^40^. According to this study, a reduction of the quantum efficiency of the phosphor by about 6% would result in a color deviation matching the outer limits of a MacAdam ellipse of step 2. This deviation continuously increases with increasing reduction of the quantum efficiency^40^. Even though, as discussed, orthosilicate based phosphors suffer from a comparably large drop of quantum efficiency, the related large color shifts highlight that the exact knowledge of the phosphor temperature is of huge relevance, also for phosphors which show a lower drop of quantum efficiency.

Albeit the present study was performed by using a CCE having specific geometrical dimensions and a specific concentration of phosphor particles in a silicone matrix, the findings presented here are of general nature, in particular for CCEs which consist of a phosphor-silicone slurry. For smaller phosphor layer heights and/or increased phosphor concentration (higher thermal conductivity) temperatures within a CCE are lower, but still show a pronounced gradient^20^, while the shape of the temperature distribution may be a bit different^16^. The same is also true for different geometries of the CCEs, like a globe-top configuration, for which, the highest temperatures have also been reported to occur within the volume^19, 41^ or for variations of the optical properties of the phosphor. For instance, a variation of the extinction coefficient of the phosphor also has some relevance on the shape of the temperature gradient^42^. On the other hand, it has to be mentioned that these temperature gradient related effects are a bit less relevant (again mostly independent from geometry and size) in case that a CCE with a high overall thermal conductivity is used, e.g. a phosphor ceramic. In this case, the thermal gradient is much weaker^43^, at least as long as also the gluing layer for the phosphor ceramic has a high thermal conductivity. Anyhow, even in case of a comparably low thermal load, phosphor thermometry can be generally applied for experimental studies on the phosphor temperature of those regions inside the CCE from which most of the converted light is emitted in an LED under operation. Using the reference curve as shown in Fig. 8, a broad range of potential phosphor temperatures can be covered. Even in case of perfect heat dissipation from the CCE, one can apply phosphor thermometry at least to ensure that the phosphor and its emission do not suffer from notable thermal impacts upon LED operation.

## Conclusions

The iterative optical and thermal simulation procedure presented here allows the determination of the temperature distribution in the CCE of a phosphor converted LED with good accuracy. From such a simulation both the highest temperatures (which occur close to the CCE surface and which are most relevant with respect to materials degradation) as well as the temperatures which are most relevant for those phosphor particles which mainly contribute to converted light emission can be determined. While infrared thermography only gives information on the surface temperature, phosphor thermography turned out as a useful tool with respect to the open question of experimental determination of the phosphor temperature of those regions inside the CCE which mainly contribute to color conversion. The latter temperature can act as input parameter for optical simulations in order to get at an estimation of expectable color shifts of phosphor converted LEDs.
